# Cardiorenal Syndrome in COVID-19 Patients: A Systematic Review

**DOI:** 10.3389/fcvm.2022.915533

**Published:** 2022-06-28

**Authors:** Ling Lin, Yangqin Chen, Dongwan Han, Andrew Yang, Amanda Y. Wang, Wenjie Qi

**Affiliations:** ^1^Department of Infectious Disease, Beijing Friendship Hospital, Capital Medical University, Beijing, China; ^2^Department of General and Acute Care Medicine, Royal North Shore Hospital, Sydney, NSW, Australia; ^3^Concord Clinical School, The University of Sydney, Sydney, NSW, Australia; ^4^Division of the Renal and Metabolic, George Institute for Global Health, TheUniversity of New South Wales, Sydney, NSW, Australia; ^5^Department of Renal Medicine, Concord Repatriation General Hospital, Concord, NSW, Australia

**Keywords:** cardiorenal syndrome (CRS), COVID-19, SARS-CoV-2, cardiac complications, renal complications

## Abstract

**Aims:**

To perform a systematic review assessing the clinical manifestations and outcomes of cardiorenal syndrome or the presence of both cardiac and renal complications in the 2019 coronavirus disease (COVID-19) patients.

**Methods:**

All relevant studies about cardiorenal syndrome or both cardiac and renal complications in COVID-19 patients were retrieved on PUBMED, MEDLINE, and EMBASE from December 1, 2019 to February 20, 2022.

**Results:**

Our search identified 15 studies including 637 patients with a diagnosis of cardiorenal syndrome or evidence of both cardiac and renal complications followingSARS-CoV-2 infection. They were male predominant (66.2%, 422/637), with a mean age of 58 years old. Cardiac complications included myocardial injury (13 studies), heart failure (7 studies), arrhythmias (5 studies), or myocarditis and cardiomyopathy (2 studies). Renal complications manifested as acute kidney injury with or without oliguria. Patients with cardiorenal injury were often associated with significantly elevated levels of inflammatory markers (CRP, PCT, IL-6). Patients with a diagnosis of cardiorenal syndrome or evidence of both cardiac and renal complications had more severe disease and poorer prognosis (9 studies).

**Conclusion:**

The presence of either cardiorenal syndrome or concurrent cardiac and renal complications had a significant impact on the severity of the disease and the mortality rate among patients with COVID-19 infection. Therefore, careful assessment and management of potential cardiac and renal complications in patients with COVID-19 infection are important to improve their outcomes.

## Introduction

The 2019 coronavirus disease (COVID-19) is an infectious disease caused by severe acute respiratory syndrome coronavirus 2 (SARS-CoV-2). Current literature indicates that sepsis secondary to COVID-19 infection has typical pathophysiological characteristics, namely early cytokine storms and subsequent immunosuppressive stages (1). Sepsis is frequently associated with cardiovascular complications and acute kidney injury either in isolation or in combination (2).

Angiotensin-converting enzyme 2 (ACE-2) is thought to be the major cell entry receptor for SARS-CoV-2 (3). ACE-2 is also expressed in the heart and kidney, providing a link between coronavirus infection and potential cardiovascular and renal complications (4). A recent epidemiological study (5) demonstrated that acute myocardial injury, cardiac arrhythmias, and shock can occur in 7.2, 18.7, and 8.7% of COVID-19 patients, respectively. Renal involvement is also not uncommon in the course of COVID-19. More than 40% of patients admitted to hospitals with COVID-19 infection had proteinuria (6). Among critically ill patients, acute kidney injury (AKI) is common, affecting ~20–40% of patients infected with COVID-19 admitted to intensive care units (7).

Although COVID-19 is most commonly associated with COVID pneumonitis, it can also result in several extrapulmonary manifestations, such as thrombotic complications, acute cardiac injury (ACI), acute kidney injury (AKI), gastrointestinal symptoms, and hepatocellular injury (8).

Cardiorenal syndrome can occur in COVID-19 patients, precipitated by arrhythmias, ACI, and AKI (2). Cardiorenal syndrome comprises a spectrum of disorders involving both the heart and kidneys, in which acute or chronic dysfunction in one organ may induce acute or chronic dysfunction in the other (9).

Limited data is available when evaluating the outcomes of COVID-19 patients with cardiorenal syndrome. Thus, the objective of this systematic review is to analyze and summarize the available literature on COVID-19 patients with both cardiac and renal complications, or cardiorenal syndrome, to gain an improved understanding of these issues in COVID-19 patients.

## Methods

### Search Strategy

The literature search was conducted in PUBMED/MEDLINE and EMBASE databases from December 1, 2019 to February 20, 2022 using the following terms: (COVID-19 OR SARS-CoV-2 OR severe acute respiratory syndrome coronavirus 2) AND (acute kidney injury OR acute renal impairment OR acute renal failure OR renal replacement therapy) AND (cardiomyopathy OR CMP OR cardiomyopathies OR myocardiopathy OR cardiac injury OR myocarditis OR heart injury) in the title/abstract. We limited our search to articles written in English. The literature search was conducted independently by three authors (LL, YQC, and DWH). Additionally, all references of selected papers were searched manually. This systematic review followed instructions from the “Preferred Reporting Items for Systematic Reviews and Meta-Analyses” (PRISMA) statement (10).

### Criteria for Inclusion

We included human studies meeting the following criteria: (1) Patients with COVID-19 were confirmed through positive results for SARS-CoV-2 nucleic acid testing of nasopharyngeal or throat swab specimens; (2) Patients 18 years or older; (3) Patients diagnosed with cardiorenal syndrome or evidence of both cardiac and renal complications. The exclusion criteria applied to the studies were: (1) Pregnant or lactating women; (2) Study type: review, conference abstract, letter to the editor.

### Data Extraction

The following variables were extracted from all included studies: first author, the country where the research was conducted, type of study, number of patients, mean age, gender, underlying comorbidities, cardiac and kidney clinical events (such as cardiac arrhythmia, cardiac injury defined as elevated troponin levels, heart failure defined as EF ≤ 40%, elevated BNP, or echocardiographic evidence of heart failure, myocarditis, oliguria, anuric, proteinuria, acute kidney injury defined as elevated serum creatinine level, tubular injury), laboratory findings, use of Angiotensin-Converting Enzyme Inhibitors (ACEI) or Angiotensin Receptor Blockers (ARB), and clinical outcomes. Three authors (LL, YQC, and DWH) independently performed data extraction. Any disagreements were discussed and resolved with the senior authors (AYW and WJQ).

## Results

**Figure 1:**
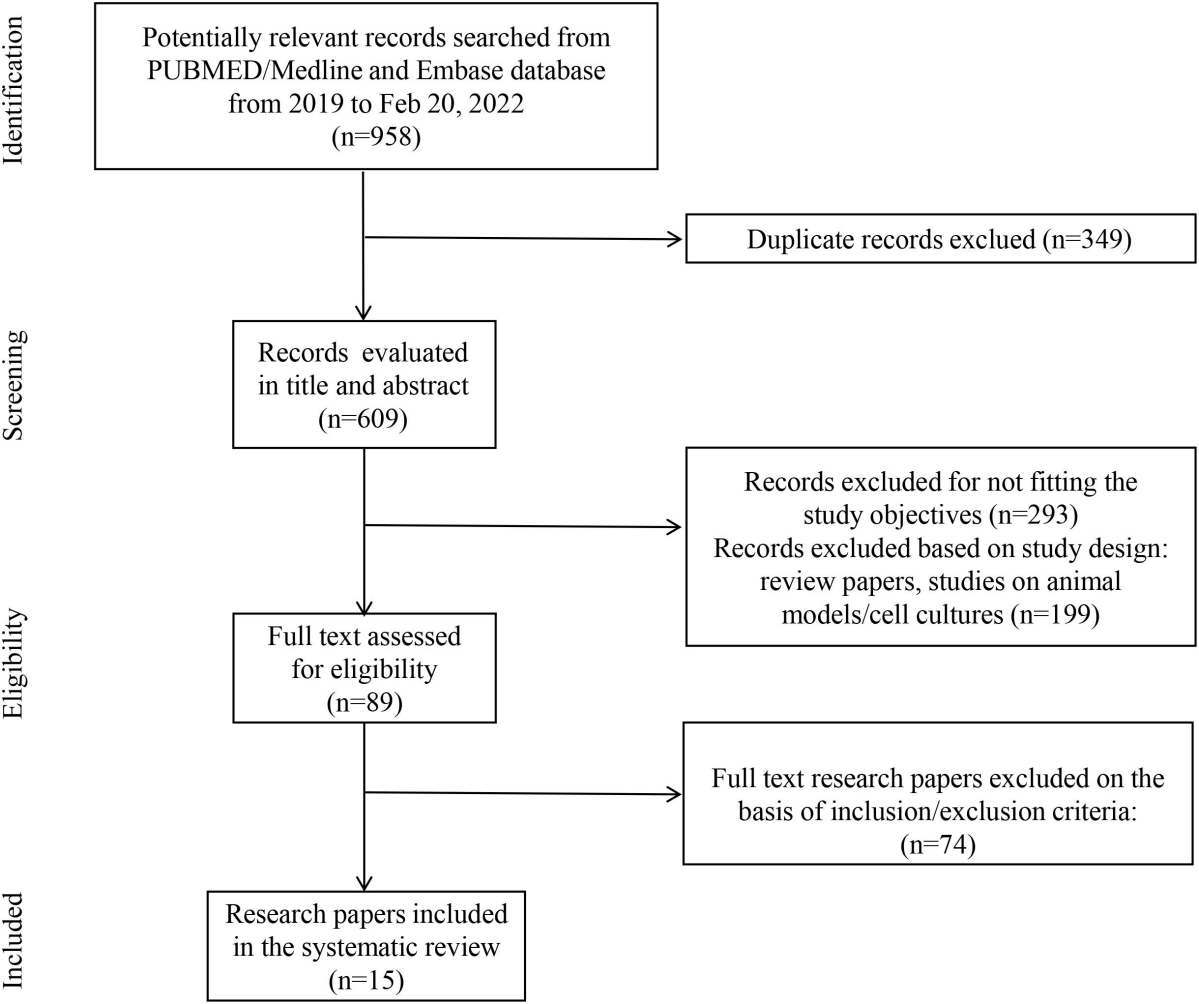
Flow diagram of the study selection process.

The search identified 15 studies and 637 patients with a diagnosis of cardiorenal syndrome or evidence of both cardiac and renal complications after SARS-CoV-2 infection. They were male predominant (66.2%, 422/637), with a mean age of 58 years old (Figure 1; Table 1).

**Table 1:** Characteristics of the included studies.

**Author**	**Country**	**Type of study**	**Total participants (n)**	**Subgroup characteristics**	**Patients with cardiac and/or renal complications (n)**	**Gender Male (%)**	**Age Mean (y)**	**Underlying diseases**	
								**Cardiovascular**	**Renal**
Ali et al. (11)	Ireland	Case report	1		1	100%	37	Cardiomyopathy?	N
Li et al. (12)	China	Retrospective study	1,249	6	6	61.9%	36	Hypertension CHD	CKD
Case et al. (13)	USA	Retrospective study	3,389	Tn↑	195	53.8%	68	47.7%Hypertension 37.4% CHD 39.5% CHF 19.0% AF	42.1%CKD
				Tn↑ with AKI	95				
				Tn N	3,194	50.9%	61	53.2% hypertension 13.1% CHD 15.1% CHF 9.6% AF	24.0%CKD
Stefan et al. (14)	Romania	Case report	1		1	0	53	Hypertension Hyperlipidemia	N
Zhu et al. (15)	China	Case report	1		1	100%	55	Hypertension CHD	Renal graft function normal
Naeem et al. (16)	United Arab Emirate	Retrospective study	203	ACI	44	91%	55	55.5% hypertension 9% cardiovascular disease	NA
				ACI and AKI	33				
				No ACI	159	70.5%	46	32.0% hypertension 3.2% cardiovascular disease	
Shi et al. (17)	China	Retrospective study	416	Tn↑	82	49.3%	74	59.8% hypertension 29.3% CHD 14.6% CHF	6.1%CKD
				Tn↑ and AKI	7				
				Tn N	334		60	23.4% hypertension 6.0% CHD 1.5% CHF	2.7%CKD
Rahimzadeh et al. (18)	Iran	Retrospective cohort study	516	AKI	194	85.1%	61	53.6% hypertension 29.4% cardiac disease	8.8%CKD 2.6%KTH
				AKI and ACI	61				
				No AKI	322	49.4%	56	33.9% hypertension 17.7% cardiac disease	0.9%CKD
Rao et al. (19)	USA	Retrospective study	8,574	No AKI	6,011	53.1%	60	52.9% hypertension 8.3% CHF 3.9% MI	8.1%CKD
				No AKI and MACE	279 (4.6%)				
				AKI Stage 1	902	62.5%	69	76.4% hypertension 18.6% CHF 5.7% MI	22.8%CKD
				AKI Stage 1 and MACE	122 (13.5%)				
				AKI Stage 2	431	63.1%	71	79.6% hypertension 15.3% CHF 6.5% MI	5.3%CKD
				AKI Stage 2 and MACE	81 (18.8%)				
				AKI Stage 3	777	64.9%	65	72.7% hypertension 12.1% CHF 4.4% MI	12.9%CKD
				AKI Stage 3 and MACE	203 (26.1%)				
Pernigo et al. (20)	Italy	Case report	1		1	100%	45	N	N
Ramalho et al. (21)	Portugal	Case report	1		1	100%	50	Dyslipidaemia	N
Saririan et al. (22)	UK	Case report	1		1	100%	61	Hypretension	N
AI-Wahaibi et al. (23)	Oman	Retrospective study	143	Tn↑	31	86.7%	61	61.3% hypretension 16.1% CHF 6.5% CHD	16.1%CKD
				Tn↑ and AKI	21				
				Tn N	112		44	24.1% hypretension 3.6% CHD	6.2%CKD
Parith et al. (24)	USA	Case report	1		1	0	23	N	N
Yasmin et al. (25)	Indonesia	Case report	1		1	0	64	N	N

*NA, Not Applicable; ?, Clinically Undetermined; IQR, Interquartile Range; SD, Standard Deviation; ACI, Acute Cardiac Injury; AKI, Acute Kidney Injury; CHD, Coronary Atherosclerotic Heart Disease; CKD, Chronic Kidney Disease; CHF, Congestive heart failure; AF, Atrial fibrillation; MACE, major adverse cardiac event; USA, The united states of America; KTH, Kidney transplant history; MI, myocardial infarction; Tn, troponin; N, Normal; N/A, Not applicable; n, Number; y, Year*.

The studies were either retrospective (7 studies) or case reports (8 studies). Most patients had multiple comorbidities including hypertension, chronic heart failure, and chronic kidney disease before SARS-CoV-2 infection, but specific data were not provided (Table 2).

**Table 2:** Clinical and laboratory findings of the heart in COVID-19 patients with cardiac and renal complications.

**References**		**Clinical events**	**Electro cardiogram**	**Echo cardiogram**	**Cardiac biomarkers**	
					**Tn (ng/L)**	**NT-proBNP (pg/mL)**
Ali et al. (11)		Heart failure	Sinus tachycardia Occasional premature ventricular	LVEF 10–15% Dilated left ventricle	N	247 (100–400)
Li et al. (12)		NA	NA	NA	>300	>2,500
Case et al. (13)	Tn↑	NA	NA	NA	2.6–13.82	NA
	Tn N				0.03–0.06	
Stefan et al. (14)		Thoracic pain	N	LVEF 45% Normal dimensions No segmentalkinetics alteration	304–889	301
Zhu et al. (15)		Heart failure Myocardial injury	Atrial fibrillation	NA	1,580	>70,000
Naeem et al. (16)		NA	NA	NA	>60	NA
Shi et al. (17)	Tn↑	13.4% chest pain	T-wave depression and inversion ST-segment depression Q waves	NA	190	1,689
	Tn N	0.9% chest pain	NA		<6	139
Rahimzadeh et al. (18)	AKI	31.4% ACI	NA	NA	10.3	NA
	NoAKI	15.5% ACI			4.3	
Rao et al. (19)	No AKI	3% cardiac arrest 4.6% MACE	NA	NA	10	215
	AKI Stage 1	9.6% cardiac arrest 13.5% MACE			100	1,223
	AKI Stage 2	13.3% cardiacarrest 18.8% MACE			110	848
	AKI Stage 3	19% cardiac arrest 26.1% MACE			100	1,490
Pernigo et al. (20)		Focal myocarditis Hypertensive Cardiomyopathy	Sinus tachycardia Left axis deviation Slight diffuse ST depression	Severe systolic and diastolic left ventricle dysfunction Myocardial thickening LVEF 30%	82	NA
Ramalho et al. (21)		Thrombus in the left ventricle Congestive heart failure	Left axis deviation	LVEF 15% Severe left ventricle dilation	1,345	30.39
Saririan et al. (22)		Myocardial ischaemia	Supraventricular tachycardia ST-elevation after adenosine	Moderate leftventricular systolic dysfunction	6,283–7,459 5,852–2,159	NA
AI-Wahaibi et al. (23)	Tn↑	12.9% atrial tachyarrhythmia 3.2% ventricular arrhythmia 9.7% bradyarrhythmia	NA	NA	NA	NA
	Tn N	0.9%Atrial tachyarrhythmia 1.8%Ventricular arrhythmia 6.5%Brady arrhythmia	NA	NA	NA	NA
Parith et al. (24)		Cardiomyopathy	A prolonged QT interval of 526 ms	Moderate global left ventricular dysfunction with an LVEF of 34% and moderate right ventricular dilatation with severe right ventricular hypokinesis	80	1,205
Yasmin et al. (25)		Cardiac injury	Fatal pulseless ventricular tachycardia	NA	420	NA

*BNP, Brain Natriuretic Peptide; Tn, Troponin; LVEF, Left Ventricular Ejection Fraction; NA, Not Applicable; N, Normal; MACE, major adverse cardiac event*.

Cardiac complications manifested as myocardial injury (13 studies), heart failure (7 studies), arrhythmia (5 studies), or myocarditis and cardiomyopathy (2 studies) (Table 2). Five studies demonstrated a reduction in left ventricular ejection fraction. Elevated troponin and brain natriuretic peptides were seen in 9 studies. Renal complications manifested as AKI with or without oliguria. However, severe AKI requiring dialysis therapy was not common (5 studies) (Table 3). Patients with cardiorenal injury were often associated with significantly elevated levels of inflammatory markers (CRP, PCT, IL-6) (Table 4). Use of ACEI/ARB occurred in 2 studies. Patients with a diagnosis of cardiorenal syndrome or evidence of both cardiac and renal complications had more severe disease and poorer prognosis (9 studies).

**Table 3:** Clinical and laboratory findings of the kidney in COVID-19 patients with cardiac and renal complications.

**Author**		**Clinical events**	**eGFR**	**Renal biomarkers**		**Dialysis**
			**(mL/min/1.73 m^**2**^)**	**Cr (μmol/L)**	**BUN (mg/dL)**	
Ali et al. (11)		Oliguria Acute tubular injury	<10	657	N	Intermittent hemodialysis
Li et al. (12)		NA	<60	NA	NA	NA
Case et al. (13)	Tn↑	48.7% AKI	58.5% ≤ 30 21.5% ≥ 60	NA	NA	NA
	Tn N	28.5% AKI	28.4% ≤ 30 55.9% ≥ 60			
Stefan et al. (14)		Oliguria Cloudy urine Proteinuria	NA	777.9	239	NA
Zhu et al. (15)		Oliguria	NA	233–308	725.4	NA
Naeem et al. (16)	ACI	75% AKI	66.5	184	NA	NA
	No ACI		94	93		
Shi et al. (17)	Tn↑	8.5% AKI	NA	101.7	NA	2.4% Continuous kidney therapy
	Tn N	0.3% AKI		56.6		0
Rahimzadeh et al. (18)	AKI	61.9% stage 1 18.0% stage 2 20.1% stage 3 63.9% proteinuria	53.48 (35.70–68.25)	118.5	44	NA
	No AKI	29.3% proteinuria		83.1	26	
Rao et al. (19)	No AKI	AKI	NA	97.3	NA	RRT
	AKI stage 1			265.2		0.6% RRT
	AKI stage 2			229.8		2.6% RRT
	AKI stage 3			618.8		36.5% RRT
Pernigo et al. (20)		AKI Acute tubular injury Hypertensive kidney disease	NA	274.1	NA	NA
Ramalho et al. (21)		AKI	NA	145.9	64	NA
Saririan et al. (22)		Anuric	NA	547.2	NA	Continuous veno-venous hemofiltration
AI-Wahaibi et al. (23)	Tn↑	67.7% AKI	NA	NA	NA	48.4% RRT
	Tn N	11.6% AKI	NA	NA	NA	3.6% RRT
Parith et al. (21, 24)		AKI	NA	198.9	NA	NA
Yasmin et al. (25)		AKI	NA	117.6	75.6	NA

*Cr, creatinine; BUN, UreaNitrogen; N, Normal; NA, Not Applicable; AKI, Acute Kidney Injury*.

**Table 4:** Inflammatory index, ACEI/ARB use and the outcomes in COVID-19 patients with cardiac and renal complications.

**Author**		**Inflammatory index**	**ACEI/ARB use**	**Outcomes (%)**
Ali et al. (11)		CRP <100 mg/L	ACEI	Cured
Li et al. (12)		PCT 0.1 ng/mL CRP 0.5–37.1 mg/L ESR 24–58 mm/h	NA	Higher mortality rate
Case et al. (13)	Tn↑	NA	NA	56.9% deceased
	Tn N			18.0% deceased
Stefan et al. (14)		CRP 2.2 mg/dL ESR 28 mm/h Ferritin 337 g/dL	NA	Cured
Zhu et al. (15)		CRP 81.6 mg/L IL-6 > 30 pg/ml	NA	Cured
Naeem et al. (16)	ACI	CRP 138.5 mg/L	NA	68.9% deceased
	No ACI	CRP 59 mg/L		5.1% deceased
Shi et al. (17)	Tn↑	CRP 10.2 mg/dL PCT 0.27 ng/mL	NA	51.2% deceased
	Tn N	CRP 3.7 mg/dL PCT 0.06 ng/mL		4.5% deceased
Rahimzadeh et al. (18)	AKI	CRP 69.4 mg/L ESR 46 mg/L	28.4%ACEI/ARB	77% severity 39.7% mortality
	Non-AKI	CRP 47.4 mg/L ESR 41 mg/L	14.3%ACEI/ARB	23% severity 7.1% mortality
Rao et al. (19)	No AKI	CRP 6.6 mg/L IL-6 23.0 pg/mL	NA	10.2% deceased
	AKI stage 1	CRP 8.1 mg/L IL-6 38.6 pg/mL		31.1% deceased
	AKI stage 2	CRP 9.1 mg/L IL-6 30.5 pg/mL		38.6% deceased
	AKI stage 3	CRP 10.0 mg/L IL-6 86.0 pg/mL		48.9% deceased
Pernigo et al. (20)		CRP 30 mg/L	NA	Cured
Ramalho et al. (21)		CRP 64.1 mg/dl	NA	NA
Saririan et al. (22)		NA	NA	Deceased
AI-Wahaibi et al. (23)	Tn↑	NA	NA	53.3% deceased
	Tn N			7.1% deceased
Parith et al. (24)		NA	NA	Deceased
Yasmin et al. (25)		PCT 0.1 ng/ml	NA	Deceased

*ACEI, Angiotensin-converting Enzyme Inhibitor; ARB, Angiotensin Receptor Blocker; NA, Not Applicable; N, Normal; CRP, C reactive protein; ESR, Erythrocyte sedimentation rate; PCT, Procalcitonin; IL, Interleukin; Tn, Troponin*.

## Discussion

Patients who developed AKI were more likely to have a cardiac event suggesting a probable role of cardiorenal interaction in the renal dysfunction that occurs in COVID-19. AKI may result in volume overload and cardiac dysfunction, and vice versa since cardiomyopathy may lead to hypotension, renal hypoperfusion, and renal congestion resulting in renal dysfunction (26), and culminating in acute respiratory distress syndrome (ARDS). The cardiorenal syndrome is associated with increased morbidity and mortality in COVID-19 patients, as well as healthcare costs.

**Figure 2:**
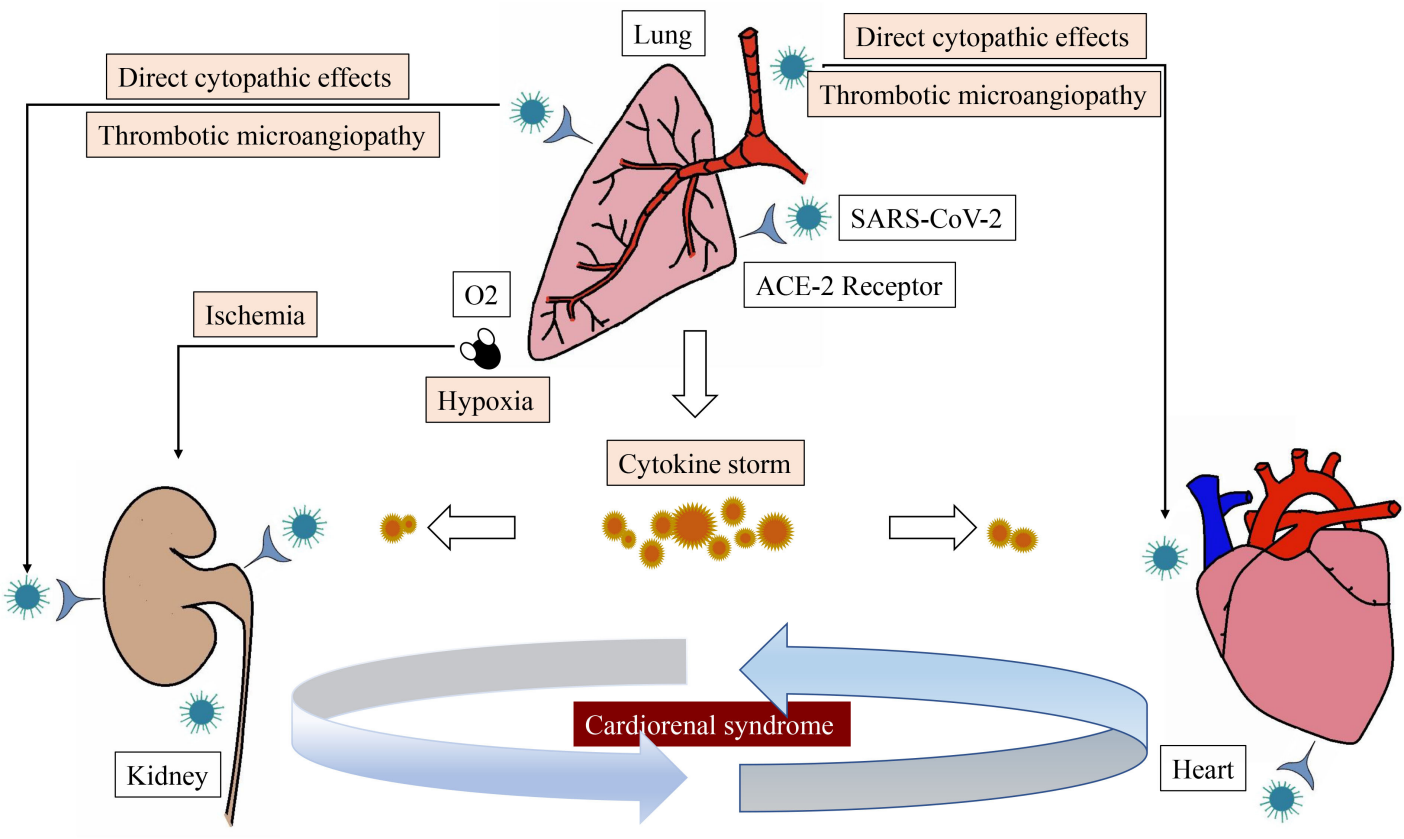
The main pathophysiological pathways of cardiorenal syndrome associated with SARS-CoV-2 infection.

COVID-19 may affect the heart and kidney through several mechanisms (Figure 2). Firstly, new evidence suggests that SARS-CoV-2 may have direct cytopathic effects on the heart and kidney. ACE-2 is the receptor for SARS-CoV-2 to enter human cells, which is highly expressed in extrapulmonary tissues including the heart and kidney (27). Secondly, excessive release of cytokines due to viral infection, known as cytokine release syndrome or cytokine storm, is the mechanism leading to multiorgan damage in COVID-19. The presence of cytokine storms and pneumonia-related hypoxia can contribute to myocardial and renal ischemia due to changes between oxygen supply and demand. Furthermore, Li et al. (28) has reported that the kinetic changes of cytokines correlate with the prognosis of patients with severe COVID-19. Thirdly, thrombotic microangiopathy seen in COVID-19 may also lead to ACI and AKI. Systemic coagulation dysfunction appears to promote thrombosis with the observation of arterial events in patients with COVID-19, such as renal artery thrombosis or acute coronary syndrome.

Up to a fifth of COVID-19 patients have an acute myocardial injury (12–17% of cases) (29, 30). In patients with SARS-CoV-2 infection, the most common features of myocardial injury were ECG changes and elevated troponin. Echocardiography showed subclinical left ventricular diastolic dysfunction and even decreased ejection fraction (EF) in severe cases (5). As previously seen during coronavirus outbreaks, patients with a low EF are more likely to require mechanical ventilation (31). This is clinically important for hospitalized patients, as expert consensus recommends an early assessment and continuous cardiac monitoring to identify patients with cardiac injury and help predict further COVID-19 complications (32). High-sensitivity troponin is a useful cardiac monitoring tool in COVID-19. Zhou et al. (30) observed a gradual increase in high-sensitivity cardiac troponin I (hs-cTnI) levels in non-survivors (reaching the reference limit on day 11), while hs-cTnI levels in survivors remained low. Piccioni et al. (33) also identified that in patients with COVID-19, high-sensitivity troponin was a negative prognostic indicator. Increased cTnI levels may be associated with endotoxin production, which may be secondary to sepsis, an overall pro-inflammatory state, or direct myocardial infarction through ACE2 receptors in cardiac tissue (34). The increase of IL-6 was parallel to that of hs-cTnI, which increased the possibility of reflecting viral myocarditis. Existing data from China show that one-quarter to one-third of COVID-19 patients have severe heart failure. Zhou et al. (30) reported 23% of heart failure in their series of 191 patients with SARS-CoV-2, while Chen et al. (35) reported 27.5% (33/120) of increased N-terminal pro-B type natriuretic peptide (NT-proBNP).

Although early reports showed a low incidence of AKI (3–9%) among COVID-19 patients in a Chinese population (5), recent data has shown a higher incidence of renal abnormalities. The most prominent findings are proteinuria or hematuria. The most significant findings were albuminuria or hematuria, which was found by test paper evaluation in nearly one-third of patients on the first day of admission, and elevated serum creatinine and blood urea nitrogen in 15.5 and 14.1% of patients (6). Importantly, an elevation of any marker of kidney damage in COVID-19 patients is associated with significantly higher hospital mortality. Several mechanisms may contribute to the kidney injury seen with COVID-19. Other mechanisms that have been reported include sepsis, acute tubular necrosis caused by renal hypoperfusion, cytokine storm, alveolar injury caused by renal medulla hypoxia, cardiorenal syndrome, and rhabdomyolysis (26, 36–38). Magoon et al. has reported less common conditions such as immune-mediated glomerulonephritis and primary glomerular lesions that caused focal segmental glomerulosclerosis collapse (39). Moreover, the hypercoagulable state in COVID-19 may lead to thrombotic microangiopathy and peritubular and glomerular capillary obstruction (38, 40). AKI may also be the result or complication of COVID-19 treatment. Antiviral drugs can lead to tubulointerstitial diseases (41, 42), and biopsy confirmed oxalate nephropathy associated with vitamin C has been reported (43). Certain antibiotics/antibacterial agents have also been implicated in AKI in COVID-19 patients (44).

ACE-2 is the main entry point of most coronaviruses, and its binding domain has a high affinity with SARS-CoV-2. The coronavirus binds to the extracellular domain of ACE-2 on the host cell surface through its spike protein (S protein), and then invades the cells, resulting in the down-regulation of ACE-2 expression on the cell surface (3). After entering cells, viruses replicate and induce cytotoxicity, which may lead to organ failure. ACE-2 is widely expressed throughout the body, with the highest expression in the gastrointestinal tract and oral epithelium, and is highly expressed in the lung, kidney, and heart (45–47). As mentioned, ACE-2 is highly expressed in the proximal tubule of the kidney (3), which may allow for direct viral cell damage resulting in tissue injury and renal failure (2). On a cellular level, ACE-2 is widely expressed in cardiac fibroblasts, myocardial cells, and coronary artery endothelial cells (48). The use of an ACEI or ARB for antihypertensive treatment in a rat model has been shown to increase ACE-2 gene expression, protein levels, and activity in hearts (49–51), which may increase the chance of SARS-CoV-2 infection or the severity of COVID-19. Whether these drugs can increase the expression and activity of ACE-2 protein in humans remains controversial. In the absence of convincing clinical data, most professional organizations suggest that ACEI or ARB treatment should be continued for patients with heart failure who have or have the risk of SARS-CoV-2 infection.

## Conclusions

Patients with cardiorenal syndrome or both cardiac and renal complications had a significant impact on the severity of the disease and mortality rate among patients with COVID-19. Therefore, emphasis should be placed on the risk factors for the development of cardiorenal syndrome, its pathophysiologic mechanisms, racial predilection, optimal therapy, and prevention in the COVID-19 patient population. However, there are limited data evaluating outcomes of COVID-19 patients with cardiorenal syndrome. Thus, further research in this area is needed.

## Data Availability Statement

The original contributions presented in the study are included in the article/Supplementary Material, further inquiries can be directed to the corresponding authors.

## Author Contributions

LL, AY, AW, and WQ designed the study. LL, YC, and DH performed the search, study selection, and data synthesis. LL wrote the first draft of the manuscript. AY, AW, and WQ revised the article. All authors contributed to the paper and approved the submitted version.

## Funding

WQ was supported by Capital's Funds for Health Improvement and Research (2020-2-2027), and Funding support for key clinical projects in Beijing, China. AW was supported by National Heart Foundation Vanguard Grant, Australia.

## Conflict of Interest

The authors declare that the research was conducted in the absence of any commercial or financial relationships that could be construed as a potential conflict of interest.

## Publisher's Note

All claims expressed in this article are solely those of the authors and do not necessarily represent those of their affiliated organizations, or those of the publisher, the editors and the reviewers. Any product that may be evaluated in this article, or claim that may be made by its manufacturer, is not guaranteed or endorsed by the publisher.
