# Supplementary material for: Cardiorenal Syndrome in COVID-19 Patients: A Systematic Review
Source: Front Cardiovasc Med. 2022 Jun 28;9:915533. doi: 10.3389/fcvm.2022.915533 (PMC9273837; doi:10.3389/fcvm.2022.915533)
Supplement: Supplementary file 1 [file Table_1.DOCX]

**Appendix**

**Table S1. Search strategy**

***EMBAS via Ovid***

1. 'coronavirus disease 2019'/exp
2. 'severe acute respiratory syndrome coronavirus 2'/exp
3. 'covid-19':ab,ti
4. 'sars-cov-2':ab,ti
5. or/1-4
6. 'acute kidney failure'/exp
7. 'renal replacement therapy'/exp
8. 'acute kidney injury':ab,ti
9. 'acute renal impairment':ab,ti
10. or/6-9
11. 'cardiomyopathy'/exp
12. 'myocarditis'/exp
13. 'cardiomyopathies':ab,ti
14. 'cardiac injury':ab,ti
15. 'myocardiopathy':ab,ti
16. ' heart injury '/exp
17. or/11-16
18. 5 AND 10 AND 17

***PUBMED/MEDLINE via Ovid***

1. 'coronavirus disease 2019'/exp
2. 'severe acute respiratory syndrome coronavirus 2'/exp
3. 'covid-19':ab,ti
4. 'sars-cov-2':ab,ti
5. or/1-4
6. 'acute kidney failure'/exp
7. 'renal replacement therapy'/exp
8. 'acute kidney injury':ab,ti
9. 'acute renal impairment':ab,ti
10. or/6-9
11. 'cardiomyopathy'/exp
12. 'myocarditis'/exp
13. 'cardiomyopathies':ab,ti
14. 'cardiac injury':ab,ti
15. 'myocardiopathy':ab,ti
16. ' heart injury '/exp
17. or/11-16
18. 5 AND 10 AND 17
